# Microscopic Difference of Hydrogen Double-minimum Potential Well Detected by Hydroxyl Group in Hydrogen-bonded System

**DOI:** 10.1038/s41598-020-61377-5

**Published:** 2020-03-11

**Authors:** Se-Hun Kim

**Affiliations:** Faculty of Science Education and Research Institute for Basic Science, Jeju National University, Jeju, 63243 Korea

**Keywords:** Materials science, Physics

## Abstract

We investigate the microscopic structure of hydrogen double-well potentials in a hydrogen-bonded ferroelectric system exposed to radioactive particles of hydrogen-ion beams. The hydrogen-bonded system is ubiquitous, forming the base of organic-inorganic materials and the double-helix structure of DNA inside biological materials. In order to determine the difference of microscopic environments, an atomic-scale level analysis of solid-state ^1^H high-resolution nuclear magnetic resonance (NMR) spectra was performed. The hydrogen environments of inorganic systems represent the Morse potentials and wave function of the eigen state and eigen-state energy derived from the Schrödinger equation. The wave functions for the real space of the localized hydrogen derived from the approximated solutions in view of the atomic scale by using quantum mechanics are manifested by a difference in the charge-density distribution.

## Introduction

Ferroelectrics are usually classified into two types: perovskite and hydrogen-bonded ferroelectrics. ABO_3_-type perovskite ferroelectrics are usually in the ferroelectric phase at room temperature. Their ferroelectric phase-transition temperature is typically 100 °C or higher than that of hydrogen-bonded ferroelectrics. The perovskite phase transition can be clearly explained using atomic displacement, which is indicative of the ferroelectric displacive model^1,2^. In the calculation of the electronic structure of perovskites BaTiO_3_ and PbTiO_3_, the hybridization of the Ti 3*d* states and O 2*p* states are especially important. Furthermore, both the materials are ferroelastic; tetragonal BaTiO_3_ has a 1% *c*∕*a* strain, whereas tetragonal PbTiO_3_ shows a large (6%) strain. Pb 6*s* and O 2*p* states are strongly hybridized in PbTiO_3_, but Ba 5*p* does not hybridize with the valence band. The Pb-O bonding interaction and the smaller ionic radius of Pb^2+^ compared to Ba^2+^ result in the larger strain in PbTiO_3_, which slightly reduces the Pb-O bond distances. Further, the Ti-O repulsion prevents the volume from shrinking enough to stabilize the cubic phase. The Ti 3*d* eigenvalues are lower in PbTiO_3_ relative to BaTiO_3_ even for the same atomic displacements^3^. Many experimental and theoretical works have carried out on high-performance perovskite nanocrystals^4–6^.

There is another theory that the origin of ferroelectricity in oxides is driven by the crucial interplay of ionic and electronic interactions^7^. In the case of hydrogen embedded in a solid lattice, the ferroelectric phase of hydrogen-bonded ferroelectrics usually exists at temperatures below 0 °C^8^. Recently, several studies reported that organic molecular crystals such as croconic acid, H_2_C_5_O_5_, switched their polarity exhibiting ferroelectricity with a high spontaneous polarization of 21 *μ*C/cm^2^ at room temperature^9^. It was found that the large polarization originates from the collective site-to-site transfer of protons along the hydrogen-bonded networks^10^. In order to describe the ferroelectricity in these organic compounds, *ab initio* calculations such as those based on first-principle density functional theory (DFT) were performed to characterize the main contribution to polarization originating from a collective charge transfer to molecular units between donor-acceptor pairs^11^.

Hydrogen, which is the lightest element, triggers a phase of the solid-state structure depending on thermal energy. While the vibrational energy of hydrogen maintains an ordered state at a low temperature, the increase of vibration frequency contributes to a disordered state in view of the order-disorder demonstrated by the ferroelectric transition^12^. The ferroelectric transition results from a correlation between the lattice and proton order-disorder^13^. Theoretically, the ferroelectric phase-transition mechanism can occur, as in proton tunneling, displacive, the proton-lattice model, *etc*.^14^. The isotope effect that occurs when a deuteron substitutes a proton has been explained by the tunneling model, in which the marked increase in the transition temperature caused by deuteration is attributed to a decrease in the tunneling integral frequency due to the effect of mass^15^. The phase-transition temperature *T*_*c*_ is related to the isotope mass effect through the geometric modification of the hydrogen bond length^16,17^. Nelmes *et al*. have shown that the isotope effect is only slightly attributable to the deuteron mass affecting the tunneling probability, and much more to the change in the O-O separation^18–20^.

Hamiltonian models can be explained by the tunneling of protons and the correlation between the proton-lattice and lattice in the phase-transition model proposed by Bussmann *et al*.^21^. The proton-lattice Hamiltonian suggests that the coupling constant is represented by the vibrational frequency^22^. The coupling constant is related to the distance *δ* (H. . . H) of the equilibrium position of the double-well potential, which is correlated with the phase-transition temperature^23^. The ferroelectric phase-transition mechanism involves a displacive component with electronic instabilities to detect a marked change in the ^31^P isotropic chemical shift around the transition temperature *T*_*c*_^24^. From previous measured data^25^, the ^1^H isotropic chemical shift data were utilized and analyzed to investigate the electronic structure in view of the experimental technique to distinguish a subtle microscopic change in a hydrogen-bonded system. High-resolution solid-state nuclear magnetic resonance (NMR) analysis techniques can help in exploring the structure of condensed matter constructed by a periodic lattice structure or a non-periodic arrangement of atoms^26^. In this regard, we explored the eigen function for the eigen state and the eigen energy in the localized structure of KH_2_PO_4_ and the Morse potential of hydrogen atoms around heavy ions, which were calculated by the SchrÖdinger equations obtained from ^1^H high-resolution NMR data.

## Experiment

A single crystal of potassium dihydrogen phosphate (KDP) was grown by the liquid aqueous method and was cut and polished by the Crystal Bank at Pusan National University. Before taking measurements, the KDP crystal was irradiated with 1 MeV hydrogen ions to a dose of 10^15^ ions/cm^2^ and kept at room temperature for several days to enable transient defects to relax into stable forms. We performed ^1^H NMR measurements with magic-angle spinning (MAS) at 10 kHz and at room temperature by using a Bruker AVANCE II 400 NMR spectrometer operating at 400 MHz at KBSI Seoul Western Center. In order to calibrate the ^1^H high-resolution MAS-NMR data, chemical shifts were measured relative to a tetramethylsilane (TMS) solution. The high-resolution MAS-NMR measurements for KDP were performed at a Larmor frequency of 400 MHz for the nuclei of proton spin *I* = 1/2 with a spinning frequency of 10 kHz. The experimental radio-frequency (*rf*) pulse sequence yielding the free induction decay (FID) signal is as follows: 3-s recycle delay, 1.0-*μ*s 90° pulse width, 8192 data acquisition points, and 32 total acquisitions. Therefore, the ultra-fast MAS technique used here has the highest dipolar line narrowing to detect the chemical shifts precisely. The wave function and Morse potential of a hydrogen atom in the pristine KDP structure and proton-beam-irradiated one were determined using the established SchrÖdinger equation with ^1^H MAS-NMR chemical shift data.

## Theoretical Methods

The Hamiltonian of proton motion inside a hydrogen bond network can be expressed as follows:

1 $$H=\frac{{p}^{2}}{2m}+{V}_{M}(x),$$

where *m* is the proton mass, *x* is its displacement from the equilibrium position, *p* is its momentum, and *V*_*M*_(*x*) is the Morse potential.

The hydrogen atom can be considered to be connected to a spring because it undergoes back-to-back oscillation in the Morse potential *V*_*M*_(*x*).

The Morse potential *V*_*M*_(*x*) is approximately expressed as

2 $${V}_{M}(x)=2D\{\exp (-2z)\cosh 2ax-2\exp (-z)\cosh ax\},$$

where the variables *D* = 2.94 eV, *a* = 1.96 Å^−1^, *r*_0_ = 0.9 Å, and *z* = *a*(*R*/2 − *r*_0_)^27^.

A motion of the hydrogen atom within O–H...O bonds can be considered under a 1-D local Morse potential *V*_*M*_(*x*). The SchrÖdinger equation provides quantum eigen energy levels *ε*_*n*_ for a localized eigen wave function *ψ*_*n*_:

3 $$H{\psi }_{n}={\varepsilon }_{n}{\psi }_{n},$$

The SchrÖdinger Eq. (3) becomes

4 $$\begin{array}{cc} & \frac{{{\rm{\partial }}}^{2}{\psi }_{n}(x)}{{\rm{\partial }}{x}^{2}}+\frac{2m}{{\hslash }^{2}}[{\varepsilon }_{n}-2D\{\exp \left(-2a(\frac{R}{2}-{r}_{0})\right)\cosh 2ax\\  & -2\exp \left(-a(\frac{R}{2}-{r}_{0})\right)\cosh 2ax\}]{\psi }_{n}(x)=0\end{array}$$

By substituting a scaling factor, we determine a general solution to the SchrÖdinger differential equation. The scaling wave function, *ϕ*_*n*_ can be introduced by setting the space coordinate to scale by 2/a, $x\to \frac{2}{a}x$.

$${\phi }_{n}(x)={\psi }_{n}(\frac{2}{a}x),$$

By replacing the scale function with the variable substitution, ${\rm{\xi }}=8{e}^{-a(\frac{R}{2}-{r}_{0})}$, the SchrÖdinger function can be expressed as follows:

$$\frac{{{\rm{\partial }}}^{2}{\phi }_{n}(x)}{{\rm{\partial }}{x}^{2}}+\frac{2mD}{{\hslash }^{2}{a}^{2}}\left[\frac{4}{D}{\varepsilon }_{n}-\xi (2-\frac{\xi }{8})-\{{\xi }^{2}{\sinh }^{4}x-\xi (4-\xi ){\sinh }^{2}x\}\right]{\phi }_{n}(x)=0$$

Into the above equation, the following scaling factor is inserted:

5 $$\xi =8{e}^{-a\left(\frac{R}{2}-{r}_{0}\right)}.$$

Thus, the equation can be solved quantum mechanically to obtain the following eigen wave functions corresponding to the eigen state energies of the ground state and the first excited state:

6 $${\varepsilon }_{0}=\frac{D}{4}({\xi }^{2}-\xi -1),{\phi }_{0}={B}_{0}\cosh (x)\exp \left(-\frac{\xi }{2}{\sinh }^{2}x\right),$$

7 $${\varepsilon }_{1}=\frac{D}{4}(\frac{{\xi }^{2}}{8}+\xi -1),{\phi }_{1}={B}_{1}\sinh (x)\exp \left(-\frac{\xi }{2}{\sinh }^{2}x\right).$$

where *B*_1_ and *B*_2_ are the normalized coefficients of amplitude in eigen wave functions.

## Results and Discussion

**Figure 1:**
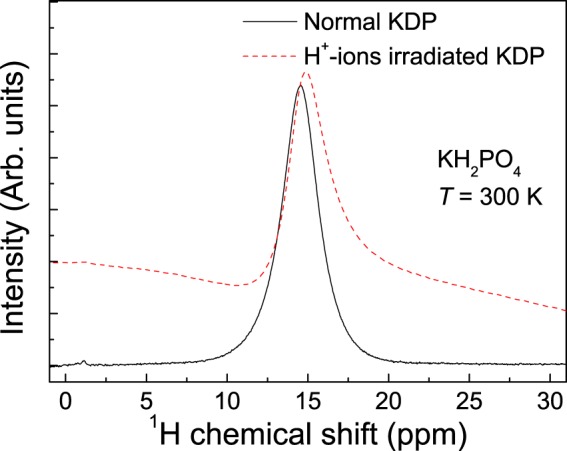
Line shape of KDP obtained from ^1^H high-resolution MAS-NMR with a spinning rate of 10 kHz at room temperature. The red dashed line corresponds to the KDP sample irradiated by H^+^ ions.

Figure 1 shows the isotropic chemical shift of the ^1^H high-resolution NMR spectrum before and after proton-beam bombardment on the KH_2_PO_4_ sample at room temperature. The peak at approximately 14.5 ppm indicates the O–H ... O bonds observed in the KH_2_PO_4_ structure at room temperature. Even though the baseline of the line shape is not flat, the spectra show distinct differences: the isotropic chemical shift is moved toward a higher frequency after the proton-beam irradiation. This analysis was also performed for comparison with the behavior of proton motions observed in previous work with the ^1^H NMR line shape analysis^28^. Proton irradiation gives rise to contrasting effects on the motions of rigid lattice protons participating in the hydrogen bond. Another ^1^H isotropic chemical shift, indicated by a peak at approximately 2 ppm, is caused by the signal of the non-hydrogen bond or “free” water OH groups^25,29^. Non-hydrated KDP samples before and after proton irradiation that did not show the signal of water were used for ^1^H MAS-NMR analysis. Further investigation is necessary to elucidate the hydrogen double-well potentials and the space distribution of hydrogen atoms in the local structure of the KH_2_PO_4_ system by using the quantum mechanical calculation in this work.

**Figure 2:**
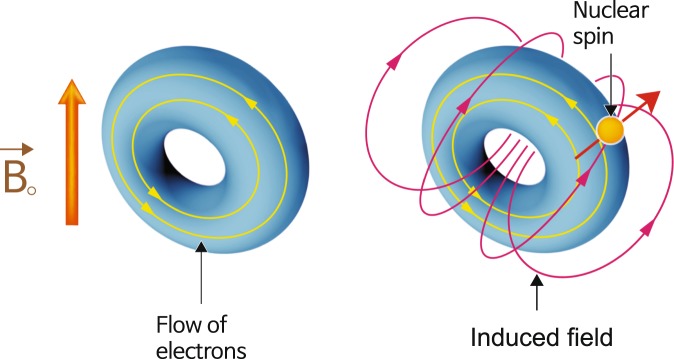
Local magnetic field around the nuclear spin, ${\overrightarrow{{\rm{B}}}}_{{\rm{local}}}=(1-\sigma ){\overrightarrow{{\rm{B}}}}_{{\rm{0}}}$^44^.

**Figure 3:**
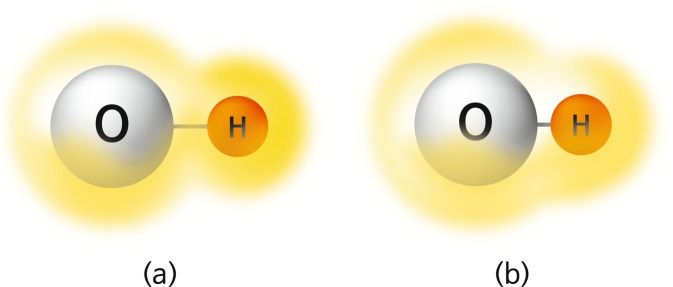
Superposition of the electron cloud around O–H along the distance between oxygen and hydrogen atoms. The electronic charge flow depends on the O–H distances (a) and (b), respectively.

The trace of isotropic chemical shifts *σ* can be obtained by the probing the hydrogen atom. The isotropic chemical shift can trace the distribution of the electron cloud around the nuclear spin. The localized field around nuclei *B*_local_ is composed of the induced field *σB*_0_ and the external field *B*_0_ by the expression *B*_local_ = (1 − *σ*)*B*_0_, as shown in Fig. 2. The isotropic chemical shift data indicate as frequency shift (ppm) in which the resonance frequency is divided by the million. An increased isotropic chemical shift implies the low field shift of paramagnetic deshielding. Oxygen atoms are covalently bonded to hydrogen atoms to form the hydroxyl group (OH–). The hydrogen atom of the hydroxyl group is weakly connected and adjacent to the other oxygen atom forming the hydrogen bond. The superposed electron cloud around hydrogen varies with the distance between the hydroxyl group and the adjacent oxygen. The electronic charge flow can be traced by the isotropic chemical shift, which indicates a hydrogen-bond change (Fig. 3). Furthermore, it can provide the electronic structure on applying quantum mechanics on the basis of experimental data. The relationship between the oxygen separation and the isotropic chemical shift is expressed by the following empirical equation.

8 $$\sigma ({\rm{p}}{\rm{p}}{\rm{m}})=79.05-255\times d({\rm{O}} \mbox{-}  \mbox{-} {\rm{H}}\cdots {\rm{O}})({\rm{n}}{\rm{m}}).$$

The related formula between the inter-oxygen distance involving the hydrogen bond (O–H ... O) and ^1^H NMR chemical shift is empirically well established by a linear function:

9 $$d({\rm{O}} \mbox{-}  \mbox{-} {\rm{H}}\cdots {\rm{O}})({\rm{\AA}})=\frac{79.05-\sigma }{25.5}$$

The inter-oxygen distance *d*(O–H ... O), (2*R*_*O*...*O*_) is reciprocally proportional to the isotropic chemical shift data^30,31^, suggesting that information on the hydrogen bond length can be obtained from the ^1^H high-resolution chemical shift data^32^.

**Figure 4:**
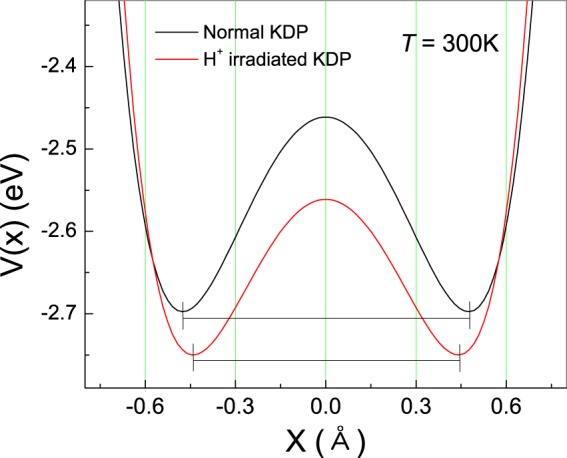
Typical double-well potentials obtained from the ^1^H high-resolution NMR line shape of KDP at 300 K. The black solid line corresponds to the pristine KDP sample, while the red dashed line corresponds to the proton-beam-irradiated KDP sample.

In order to investigate the local hydrogen environments, we can adopt a two-Morse potential depicted by a double minimum potential, as shown in Fig. 4. At *T* = 300 K, the KDP crystal is in the paraelectric phase. The disordered proton state around heavy atoms is correctly described by the symmetric double-well potential given by Eq. (2)^33,34^.

**Figure 5:**
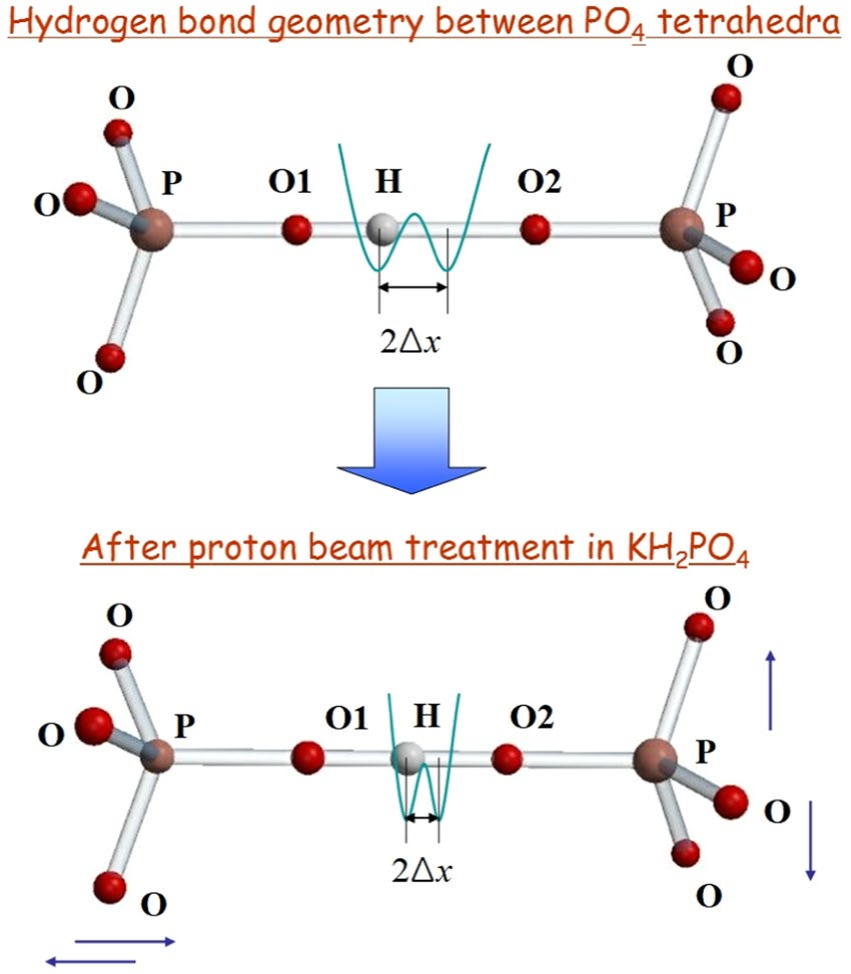
Structural modifications in the geometry of the hydrogen bond are closely related with the two equilibrium positions of protons between PO_4_ tetrahedra after the proton-beam irradiation.

Figure 4 shows the double-well potentials obtained from the ^1^H high-resolution NMR of KDP before and after the proton-beam irradiation. The depth of the potential decreased after the irradiation. Moreover, the distance between the equilibrium positions of double-minimum potential decreased from 0.9535 Å to 0.8889 Å after the proton irradiation. A previous report on dielectric spectroscopy showed that the distance between the equilibrium positions of two hydrogen sites, 2Δ*x*, obtained using a mean-field approximation decreased after proton-beam irradiation (Fig. 5)^35^.

Tunneling in the antiferroelectric-paraelectric transition with regard to the isotope effect in squaric acid was suggested on the basis of path integral molecular dynamics (PIMD) simulations^36^. The ferroelectric and paraelectric states of KDP were studied to elucidate the momentum distribution functions (MDF) of correlated proton motion by using PIMD simulations rather than experiments^37^.

**Figure 6:**
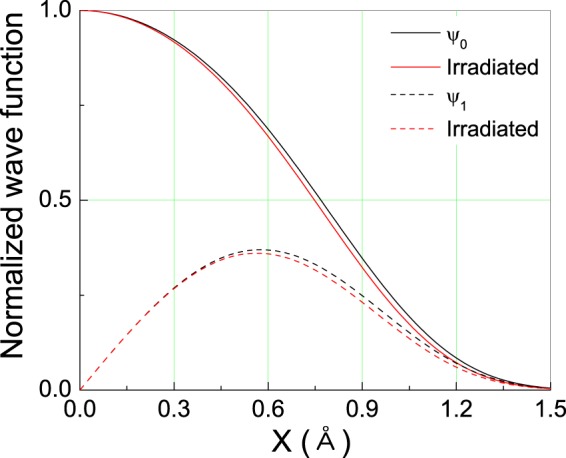
Wave functions of the ground state and the first exited state in the localized proton space distribution obtained from the ^1^H NMR line shape of KDP.

 Figure 6 shows the normalized wave function corresponding to the ground state and first excited state of protons before and after the proton-beam irradiation in the KDP system. In general, the wave functions of the ground state and first excited state described the even function and odd function in parity. The full width at half maximum (FWHM) can be considered to be proportional to the uncertainty of space distribution Δ*x* for the wave function *ψ*(*x*). The space distribution of localized protons can be described by a Gaussian distribution. The standard deviation of the space coordinate Δ*x* is compared with the FWHM. Therefore, the localized proton distribution seems to define the standard deviation of the space coordinate Δ*x*. The uncertainty of the space distribution Δ*x* corresponding to the wave function of both the ground state and the excited state, which is considered to be the separation between the two equilibrium positions in the double-well potential, slightly decreased with the proton-beam irradiation, as shown in Fig. 5. The distance between the oxygen atoms (*i.e*., O1 and O2) was reduced from 2.53 ± 9.81 × 10^−4^ Å to 2.50 ± 2.26 × 10^−3^ Å after the proton-beam irradiation using the defined formula (9). Therefore, the average distance between the two equilibrium positions of the hydrogen atoms is expected to decrease in proportion to the distance between the oxygen atoms^23^.

In an electronic structure calculation based on the density functional theory (DFT), the substitution of a deuteron for a proton weakens the proton-mediated covalent bonding, which implies a depletion of the proton probability density at the O-H-O bridge center. The DFT calculation demonstrates the difference in H/D values between the KDP samples, in which the more delocalized protons require a more attractive binding energy in their covalent bonds with oxygen^38^. The isotope effect is closely related to the geometric effect with the proton being off-center^39^. Bussmann *et al*. suggested that the phase-transition model can be explained by the proton-lattice Hamiltonian *H*_*TL*_ = *C*(*δ*)∑_*q*_*S*^*z*^*u*(*q*), where *S*^*z*^ = ± *S*^*x*^ and *u*(*q*) refers to the pseudospin for the equilibrium positions of the proton and *q*-dependent shell displacement coordinate of PO_4_. The coupling constant *C*(*δ*) is a function of *δ*, which shows the relationship between the proton and the PO_4_ tetrahedra, where *δ* is the separation of H ... H or D ... D^14,21,22^. Experimental results based on Raman spectroscopy demonstrated that the asymmetric stretching vibrational lattice mode of the D_2_PO_4_ molecule is closely related to the phase transition of K(H_1−*x*_D_*x*_)_2_PO_4_ crystals^40,41^.

The eigenfunction *ψ*(*x*) satisfies the commutation relationship [*p*, *x*]*ψ*(*x*) = −*iℏ*, where *p* and *x* are the momentum and position operators, respectively, and *ℏ* is the Planck constant^42^. The limit on observables necessitated by quantum mechanics determines the momentum and position distribution of the vibrational lattice for the soft phonon mode. A certain variable position uncertainty, Δ*x*, implies that there is a geometric change corresponding to the temperature of the ferroelectric phase transition. Previous work reported that the temperature dependence of the transverse dielectric susceptibility in deuterated KDP system showed an increasing phase-transition temperature after proton irradiation. The inverse dielectric susceptibility of the Curie-Weiss law obtained using the mean-field approximation indicates that the *μ*_2_ value ~ 2Δ*x* (separation of the proton equilibrium positions) increases from 6.29 × 10^−18^ to 6.57 × 10^−18^ cgs^43^.

In summary, we studied the microstructural changes of KDP ferroelectrics by performing the proton-beam-irradiation treatment. Measurements of highly resolved proton MAS-NMR spectra in KDP ferroelectrics were performed. From the chemical shift data, the separation between oxygens, O1-O2 were known and the equilibrium distance of hydrogen is reduced. The electron cloud of the hydroxyl group in the OHO hydrogen bond detected by the measured ^1^H isotropic chemical shift data enables the rendering of the hydrogen environment of the hydrogen bond geometry. The distance between equilibrium positions in the Morse potential decreased after the proton-beam irradiation. By introducing the scaling wave function, the exact solution of the scaled Schr$\ddot{{\rm{o}}}$dinger’s equation has been successfully calculated. Consequently, the eigen values and functions corresponding to the ground and first excited states were obtained. After the proton beam irradiation, the subtle differences were shown in the normalized wave functions of the ground and first excited states. In addition, the widths of the ground state and the first exited state of the wave function determined using the SchrÖdinger equation decreased. Therefore, our experimental results sufficiently support the theoretical results. The depiction of the localized electronic structure by using ^1^H high-resolution MAS NMR can be experimentally developed as an auxiliary tool in addition to theoretical first-principles DFT calculations.
